# *Allodia* Winnertz from the Himalayas, with nine species new to science (Diptera, Mycetophilidae)

**DOI:** 10.3897/zookeys.820.31618

**Published:** 2019-01-29

**Authors:** Trude Magnussen, Geir E. E. Søli, Jostein Kjærandsen

**Affiliations:** 1 Natural History Museum, University of Oslo, PO Box 1172, Blindern, NO-0318 Oslo, Norway University of Oslo Oslo Norway; 2 Tromsø University Museum, The Arctic University of Norway, PO Box 6050 Langnes, NO-9037 Tromsø, Norway The Arctic University of Norway Tromsø Norway

**Keywords:** Bhutan, Exechiini, fungus gnats, key, mountains, Mycetophilinae, Nepal

## Abstract

An extensive collection of fungus gnats from Nepal and Bhutan, deposited at Kyushu University Museum in Japan, has been examined and revealed nine species of *Allodia* Winnertz, 1864 new to science: *Allodiacaligata* Magnussen, **sp. n.**, *A.dibolia* Magnussen, **sp. n.**, *A.shimai* Magnussen, **sp. n.**, *A.spathulata* Magnussen, **sp. n.**, *A.horologia* Magnussen, **sp. n.**, *A.himalayensis* Magnussen, **sp. n.**, *A.nepalensis* Magnussen, **sp. n.**, *A.thudamensis* Magnussen, **sp. n.**, and *A.scalprata* Magnussen, **sp. n.** All specimens were collected at high altitudes in the central and eastern Himalayas. The species all belong to the subgenusAllodia s. str. and constitute the first records of the genus *Allodia* in Nepal and Bhutan. *Brevicornunigrofasciatum* (Brunetti, 1912) **comb. n.**, originally described from northern India, is transferred from *Allodia* based on the original description. A key for the identification of the new species is provided.

## Introduction

The Himalayas form a mountain range separating the plains of the Indian subcontinent from the Tibetan Plateau, and spread across the five countries Nepal, India, Bhutan, China, and Pakistan. It constitutes the border between the Palaearctic and the Oriental biogeographical regions, or can also be regarded as a part of, or bordering, the Sino-Japanese realm as defined in Holt et al. (2013). The Himalayas are considered a hotspot for biodiversity and the fauna constitutes a rich mixture of taxa from surrounding biogeographical regions, in addition to a large portion of endemics (e.g., Alfred et al. 2001, Huang et al. 2008, Konstantinov et al. 2009).

The fungus gnat fauna (Mycetophilidae) of the Himalayas is not properly accounted for, but the few previous studies from the region reveal a remarkable diversity with numerous species with an isolated and unique systematic position (e.g., Väisänen 1996, 2013a, b). Ten genera in the subfamily Mycetophilinae have been recorded with 31 identified species (see Table 1). As the subfamily is richly represented in both Alpine and Arctic habitats elsewhere (e.g., Kjærandsen et al. 2007), we consider this a very low number, primarily reflecting the need for further surveys.

**Table 1.** List of Mycetophilinae species recorded from the Himalayas. In cases where there are several localities within the region only the type locality is listed.

Species	Locality	Reference
***Anatella* Winnertz, 1862**		
One unidentified species	**Nepal**, Dhorpar Kharka	Kallweit and Martens 1995
***Brevicornu* Marshall, 1896**		
*B.mathei* Kallweit, 1995	**Nepal**, Dhorpar Kharka	Kallweit and Martens 1995
*B.nigrofasciatum* (Brunetti, 1912), comb. n.	**India**, Shimla and Dehradun	Brunetti 1912
***Exechia* Winnertz, 1864**		
*E.dizona* Edwards, 1924	**Nepal**, Taplejung	Kallweit and Martens 1995
*E.pararepandata* Kallweit, 1995	**Nepal**, Dhorpar Kharka	Kallweit and Martens 1995
*E.basilinea* Brunetti, 1912	**India**, Kurseong	Brunetti 1912
*E.semifumata* (Brunetti, 1912)	**India**, Darjiling	Brunetti 1912
***Epicypta* Winnertz, 1864**		
*E.cinctiventris* (Brunetti, 1912)	**India**, Simla	Brunetti 1912
*E.griseolateralis* (Brunetti, 1912)	**India**, Darjeeling	Brunetti 1912
***Macrobrachius* Dziedzicki, 1889**		
*M.longicosta* Brunetti, 1912^†^	**India**, Kurseong	Brunetti 1912
***Mycetophila* Meigen, 1803**		
One unidentified species of the *ruficollis* group^‡^	**India**, Darjeeling and Shimla	Brunetti 1912
*M.curvilinea* Brunetti, 1912	**India**, Darjiling	Brunetti 1912
*M.franzi* Plassmann, 1977	**Nepal**, Jumla	Plassmann 1977
*M.fungorum* (De Geer, 1776)	**Nepal**, Walungchung Gola	Kallweit and Martens 1995
*M.irregularis* Kallweit, 1995	**Nepal**, Dhorpar Kharka	Kallweit and Martens 1995
*M.magnicauda* Strobl, 1995	**Nepal**, Omje Khola	Kallweit and Martens 1995
*M.extincta* Loew, 1869	**Nepal**, Omje Khola	Kallweit and Martens 1995
*M.ocellus* Walker, 1848	**Nepal**, Omje Khola	Kallweit and Martens 1995
*M.quadrifasciata* Brunetti, 1912	**India**, Shimla	Brunetti 1912
*M.ruficollis* (Zetterstedt, 1852)	**Nepal**, Taplejung	Kallweit and Martens 1995
*M.suffusa* Brunetti, 1912	**India**, Shimla	Brunetti 1912
*M.taplejungensis* Kallweit, 1995	**Nepal**, Dhorpar Kharka	Kallweit and Martens 1995
***Phronia* Winnertz, 1864**		
*P.arisaema* Kallweit, 1995	**Nepal**, Dhorpar Kharka	Kallweit and Martens 1995
*P.bicuspidalis* Kallweit, 1995	**Nepal**, Dhorpar Kharka	Kallweit and Martens 1995
***Pseudexechia* Tuomikoski, 1966**		
*P.macrocantha* Kallweit, 1995	**Nepal**, Dhorpar Kharka	Kallweit and Martens 1995
***Pseudobrachypeza* Tuomikoski, 1966**		
*P.floralis* Kallweit, 1995	**Nepal**, Nepal-Himalaya	Kallweit and Martens 1995
***Rymosia* Winnertz, 1864**		
*R.albolateralis* Brunetti, 1912	**India**, Naini Tal	Brunetti 1912
***Trichonta* Winnertz, 1864**		
*T.contenta* Gagné, 1981	**Nepal**	Gagné1981
*T.fidelis* Gagné, 1981	**Nepal**	Gagné1981
*T.flebilis* Gagné, 1981	**Nepal**	Gagné1981
*T.sobria* Gagné, 1981	**Nepal**	Gagné1981
*T.superba* Gagné, 1981^§^	**Nepal**	Gagné1981
*T.genitalis* (Brunetti, 1912)^|^	**India**, Darjiling	Brunetti 1912
***Zygomyia* Winnertz, 1864**		
One unidentified species	**Nepal**, Dhorpar Kharka	Kallweit and Martens 1995

^†^Edwards (1924) indicated that this could actually be a species of *Exechia* s. l., but the prolonged costa would not be in accordance with any known *Exechia*, rather conforming with *Brachyradia* Ševčík & Kjærandsen, 2012. ^‡^ as *M.binotata* Brunetti, 1912 preocc. Colless and Liepa (1973) used the name *M.lineola*, which applied to all species of the *M.ruficollis* group. This species should therefore be regarded as an unidentified species in the *M.ruficollis* group. ^§^ Junior primary homonym. ^|^ as *Rhymosia*.

The genus *Allodia* Winnertz, 1864 is one of 20 genera in the tribe Exechiini (Ševčík and Kjærandsen 2012), and is further split into two morphologically distinct sub-genera; *Allodia* s. str. and *Brachycampta* Winnertz, 1864. The two subgenera are best separated based on the outline of the male genitalia but differ also in the arrangement of the discal bristles, in the wing venation and in the abdominal colour markings (Zaitzev 2003). The genus, as presently known, reveals a predominantly Holarctic distribution, with most of the 88 described species originating from the Palaearctic (e.g., Zaitzev 2003) and the Nearctic (e.g., Zaitzev 1983) regions. Bechev (1999), however, characterised *Allodia* as sub-cosmopolitan as some species have been described from the Afrotropical (Matile 1978), Oriental (Brunetti 1912, Edwards 1928, Senior-White 1922), and Oceanian regions (Colless 1966). Furthermore, Magnussen et al. (2018) described additional six new species of *Allodia* s. str. from the Afrotropical region.

As part of a study dealing with the systematics of the genus *Allodia*, we have examined a collection of *Allodia* s. str. from Nepal and Bhutan, which forms the basis of the present work.

## Materials and methods

During a short-term visit to Japan in 2011 one of the authors (JK) was given the opportunity to go through and sort out a substantial collection of Exechiini from a large collection of fungus gnats at the Kyushu University Museum. The material was collected in Japan and several other Asian countries. This material had been pinned, sorted to genera, and is still retained at Tromsø University Museum as a long-term loan. One substantial part of the material was collected during the Kyushu University scientific expedition to the Nepal Himalaya in 1971 and 1972. Additional material was collected by U Emoto in east Nepal in 1981. The majority, however, originates from the private collection of T Saigusa, including materials from his collecting trip to Bhutan in 1993. All primary types and most of the materials will be returned and deposited at Kyushu University Museum, Japan (**KUEC**), while some paratypes will be kept and deposited at Tromsø University Museum, Norway (**TMU**).

The *Allodia* material studied here consists of 39 males and 89 females, all pinned. These specimens were collected from various localities in Nepal and Bhutan between 1971 and 1993, at altitudes ranging from 1900 to 4000 metres above sea level. As few, if any, reliable species specific characters were found except those in the male genitalia, no further attempts were made to associate the sexes, and females are omitted from the study. The clearest differences between the male specimens were found in the outline of the gonostylus. Consequently the gonostylus has been figured, from the inner side, for straightforward comparison of the species.

The specimens were examined under light microscope. The terminalia of the males were removed, and subsequently macerated in warm lactic acid. Dissections and temporary slides were made in glycerine. The slides were studied with a Zeiss Axio imager M2, and photographed with the camera ‘Axiocam 506 color' fitted to the microscope. Based on photographs, drawings were made in Adobe Illustrator. The terminalia were afterwards transferred to glycerine in microvials and stored together with the associated pinned specimens.

**Figure 1.**
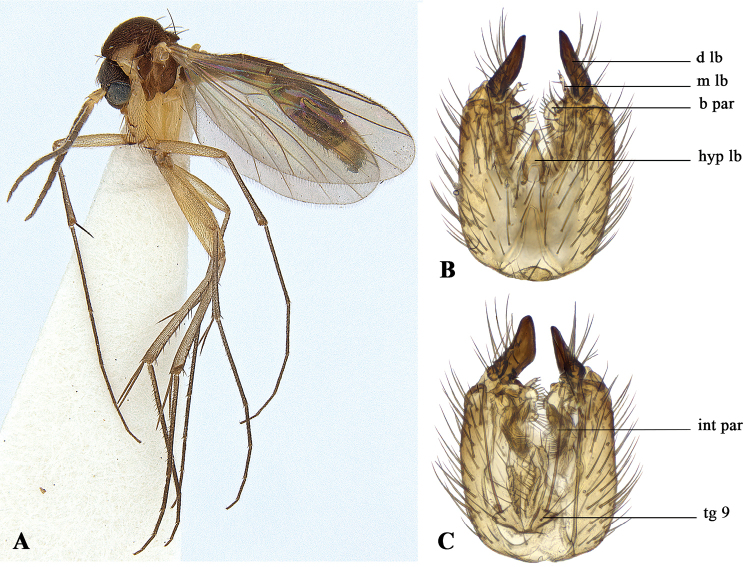
*Allodiacaligata***sp. n. A** habitus **B** genital capsule, ventral view **C** genital capsule, dorsal view. Abbreviations: **b par** = basal part, **d lb** = dorsal lobe, **hyp lb** = hypandrial lobe, **int par** = internal part, **m lb** = median lobe, **tg** = tergite.

**Figure 2.**
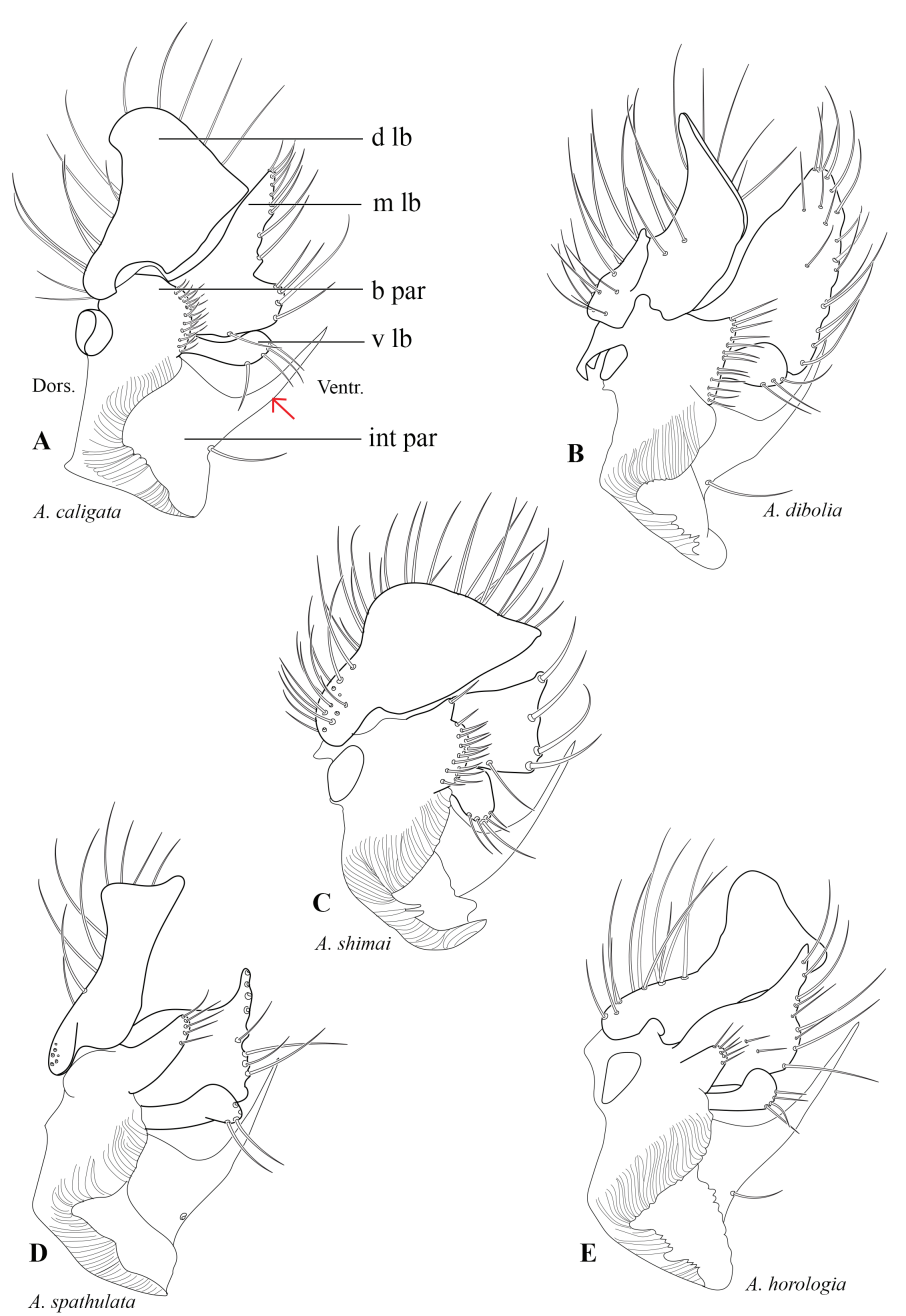
Gonostyli from inner side. **A***Allodiacaligata***B***Allodiadibolia***C***Allodiashimai***D***Allodiaspathulata***E***Allodiahorologia*. Abbreviations: **b par** = basal part, **d lb** = dorsal lobe, **int par** = internal part, **m lb** = median lobe, **v lb** = ventral lobe. Red arrow indicates the caudally projecting process of the internal lobe.

The general terminology is mainly in accordance with Søli (1997). The terminology used to describe and name the different structures of the male terminalia is shown in Figs 1B, C, and 2A, and described in detail in Magnussen et al. (2018).

### Key to the *Allodia* species of Nepal and Bhutan

The key is restricted to males, as only males could be satisfactory separated by morphological characters. The terminology used in the description of the male genitalia is explained in Figs 1B, C, and 2A.

**Table:** 

1	Internal part of gonostylus with caudally projecting process (Fig. A)	**2**
–	Internal part of gonostylus without caudally projecting process (Fig. 3A)	**6**
2	Median lobe of gonostylus with more than four setae apically (Fig. 2A)	**3**
–	Median lobe of gonostylus with four setae apically (Fig. 2C)	*** A. shimai ***
3	Basal part of gonostylus sessile with more than six setae	**4**
–	Basal part of gonostylus projected with less than six setae	**5**
4	Dorsal lobe of gonostylus sharply pointed apically (Fig. 2B)	*** A. dibolia ***
–	Dorsal lobe of gonostylus smoothly rounded apically (Fig. 2A)	*** A. caligata ***
5	Basal part of gonostylus less than half the length of median lobe (Fig. 2E)	*** A. horologia ***
–	Basal part of gonostylus half the length of median lobe (Fig. 2D)	*** A. spathulata ***
6	Dorsal lobe of gonostylus truncated apically (e. g. 3D)	**7**
–	Dorsal lobe of gonostylus rounded apically (Fig. 3B)	*** A. nepalensis ***
7	Dorsal lobe of gonostylus with dorsal hook apically (e. g. Fig. 3C)	**8**
–	Dorsal lobe of gonostylus with rounded dorsal corner apically (Fig. 3D)	*** A. scalprata ***
8	Basal part of gonostylus projected (Fig. 3A)	*** A. himalayensis ***
–	Basal part of gonostylus sessile, merged with internal part (Fig. 3C)	*** A. thudamensis ***

## Taxonomy

### Allodia caligata

Taxon classificationAnimaliaDipteraMycetophilidae

Magnussen sp. n.

http://zoobank.org/E7B31A33-3A4A-4169-AC3A-CACE152A9FD6

Figs 1 , 2A

#### Diagnosis.

The dorsal lobe of the gonostylus is distinctly boot-shaped, and together with the slightly bifurcated shape of the median lobe, *A.caligata* can be separated from other described *Allodia* species. Moreover, the species is small (< 2.7 mm) and has a distinctive colouration of scutum, overall dark, but with yellow humeri (Fig. 1).

#### Type locality.

**NEPAL**: Province no. 1 (Kosi Zone), Terhathum District, Basantapur, 2300 m a.s.l.

#### Type specimens.

***Holotype: *** male. 3 printed labels: (**E. NEPAL**) Basantapur (2300 m) 27°06'N, 87°23'E-27°08'N, 87°26'E / May 6, 1972 J. Emoto leg. Kyushu Univ. Col. / TSZD-JKJ-104928 (KUEC; pinned with genitalia in separate microvial). ***Paratypes***: **NEPAL**: Same data as for holotype, TSZD-JKJ-104939, male (KUEC), TSZD-JKJ-104940, male (KUEC); **NEPAL**: Gandaki Pradesh, Myagdi District, Dobang Kharka, 28°36'N, 083°24'E, 2400 m a.s.l., Malaise trap, 14-15 Oct 1971, leg. A. Nakanishi, TSZD-JKJ-104930, male (TMU); 14 Oct 1971, leg. A. Nakanishi, TSZD-JKJ-104931, male (KUEC); 20 Oct 1971, Leg. A. Nakanishi, TSZD-JKJ-104932, male (KUEC), TSZD-JKJ-104933, male (KUEC); 23 Oct 1971, Leg. A. Nakanishi, TSZD-JKJ-104934, male (KUEC); Malaise trap, 24-28 Oct 1971, Leg. A. Nakanishi, TSZD-JKJ-104935, male (KUEC), TSZD-JKJ-104936, male (KUEC); 28 Oct 1971, Leg. A. Nakanishi, TSZD-JKJ-104937, male (KUEC); 30 Oct 1971, Leg. A. Nakanishi, TSZD-JKJ-104938, male (KUEC); **NEPAL**: Sankhuwasabha District, Topke Gola along trail to Thurukba, 27°38'N, 087°35'E, 2600–3700 m a.s.l, 12 Jun 1972, Leg. H. Shima, TSZD-JKJ-104941, male (KUEC); 9 Jul 1972, Leg. P. Norbu, TSZD-JKJ-104942, male (KUEC); **BHUTAN**: Thimpu District, East of Dochhu La, 27°29'16”N, 089°45'44”E, 2800 m a.s.l, 2 Aug 1993, Leg. T. Saigusa, TSZD-JKJ-104929, male (TMU).

#### Description.

Body length 2.2–2.7 mm; wing length 2.4–2.7 mm. **Colouration.** Head and clypeus brown. Mouthparts and palpomeres yellow. Antennae brown, with scape, pedicel, and first half of first flagellomere whitish yellow. Scutum brown, with humeri yellow. Antepronotum yellow, other lateral sclerites brown. Wings clear without markings. Halteres whitish yellow. Legs whitish yellow. Abdomen brown, tergites II-IV with lateral area yellow, larger towards posterior margin, in some specimens only barely visible. Terminalia yellow. **Head**. Three ocelli, median ocellus smaller than laterals; lateral ocelli touching eye margin. Head covered with fine trichia. Antennae almost twice as long as thorax. Scape and pedicel with several setae on distal third. Flagellomeres cylindrical, densely clothed with fine trichia. First flagellomere twice as long as pedicel. **Thorax.** Antepronotum with four strong setae. Scutum covered with uniform small, pale setae; strong prealar and postalar setae. Discal bristles absent. Scutellum with two strong bristles. Laterotergite with few shorter setae. Other lateral sclerites bare. **Legs.** All tibiae with short setae arranged in rows. Mid tibia with six long anterodorsal and on distal 3/4 of segment numerous short posterodorsal bristles present. Hind tibia with eight anterodorsal and six posterodorsal bristles. **Wings**. Sc short, ending in R. Length of rm equal to stem of posterior fork. Base of anterior fork clearly before base of posterior fork. R1 with distal setae, R5 without. **Male terminalia**. Tergite IX medially divided, each part rounded, covered with minute trichia, with one strong apical bristle and several small setae. Hypandrial lobe heavily sclerotised and elongated. Gonostylus with dorsal lobe boot-shaped, outer surface with numerous strong setae, denser towards basis. Median lobe with strong setae along posterior border, medially with shallow incision, posterodorsal part pointed, posteroventral part more rounded; ventral border with one long seta situated medially. Ventral lobe elongated, club-shaped, with four strong setae at apex. Basal part of gonostylus blunt, with several short setae along edge. Internal part of gonostylus with long, caudally projecting process.

#### Etymology.

From Latin, *caligatus*, booted. Named after the shape of the dorsal lobe of the gonostylus.

#### Remarks.

The boot-shaped dorsal lobe and the bifurcated median lobe of the gonostylus make *A.caligata* very distinct. It cannot be confused with, any of the other species described here, or to other *Allodia* species already described or recorded from the Palaearctic.

### Allodia dibolia

Taxon classificationAnimaliaDipteraMycetophilidae

Magnussen sp. n.

http://zoobank.org/E3993CB8-1D0D-4A7F-A91A-B4F9625502DC

Fig. 2B

#### Diagnosis.

The dorsal lobe of the gonostylus is tapering towards apex, and the median lobe is distinctly flattened and broad. Additionally, the basal lobe of the gonostylus is large, flat with many thick, short setae. All studied specimens of *A.dibolia* are small (< 2.7 mm), compared to other species described from the Himalayan range. The specimens are all fairly uniform in their colouration patterns, but some are darker than others.

#### Type locality.

**NEPAL**: Province no. 1 (Kosi Zone), Terhathum District, Basantapur, 2300 m a.s.l.

#### Type specimens.

***Holotype*** : male. 3 printed labels: (**E. NEPAL**) Basantapur (2300 m) 27°06'N, 87°23'E---27°08'N, 87°26'E / May 6, 1972 J. Emoto leg. Kyushu Univ. Col. / TSZD-JKJ-104944 (KUEC; pinned with genitalia in separate microvial). ***Paratypes***: **NEPAL**: Province no. 1 (Kosi Zone), Terhathum District, Basantapur, 27°06'N, 087°23'E, 2300 m a.s.l, 29 Apr 1972, Leg. H. Shima, TSZD-JKJ-104946, male (KUEC), TSZD-JKJ-104947, male (TMU); 9 May 1972, Leg. H. Shima, TSZD-JKJ-104948, male (KUEC); **NEPAL**: Bhojpur District, Chiaksila, 27°26'N, 086°57'E, 2730 m a.s.l, 16 Jul 1981, Leg. J. Emoto, TSZD-JKJ-104949, male (KUEC); **NEPAL**: Sankhuwasabha District, Salpa La, 27°27'N, 086°55'E, 3000–3050 m a.s.l, 29 Jul 1981, Leg. J. Emoto, TSZD-JKJ-104950, male (KUEC); **NEPAL**: Province no. 3 (Nepalmandal, Bagmati), Nuwakot District, Sheopuri Lekh (= Siwapuri), Shivpuri Nagarjun NP, 27°48'N, 085°22'E, 2460 m a.s.l, 28 May 1981, Leg. J. Emoto, TSZD-JKJ-104951, male (KUEC); **NEPAL**: Province no. 3 (Sagarmatha Zone), Solukhumbu District, Junbesi Khola, 27°36'N, 086°33'E, 3400–3500 m a.s.l, 12 Aug 1981, Leg. J. Emoto, TSZD-JKJ-104952, male (KUEC); **BHUTAN**: Paro District, Jilay La, 27°22'11”N, 089°20'47”E, 3800 m a.s.l, 19 Aug 1993, Leg. T. Saigusa, TSZD-JKJ-104945, male (TMU).

**Description.** Body length 2.2–2.7 mm; wing length 2.2–2.8 mm. **Colouration.** Head and clypeus dark brown. Mouthparts, including palpomeres yellow. Antennae brown, with scape, pedicel, and base of first flagellomere yellow. Scutum brown, with narrow yellow lateral area, from humerus towards wing base. Antepronotum yellow, other lateral sclerites brown. Wings clear without markings. Halteres yellow. Legs yellow. Abdomen brown, tergites II-IV with whitish yellow area, larger towards posterior margin. Terminalia yellow. **Head.** Three ocelli present, median ocellus smaller than laterals, lateral ocelli touching eye margin. Head covered with fine trichia, except for few setae near eye margin. Antennae approx. twice as long as thorax. Scape and pedicel with several setae on distal third. Flagellomeres cylindrical, densely clothed with fine trichia. First flagellomere almost twice as long as pedicel. **Thorax.** Antepronotum with four strong setae. Scutum covered with uniform small, pale setae; strong prealar and postalar setae. Discal bristles absent. Scutellum with two strong bristles. Laterotergite with three short setae. Other lateral sclerites bare. **Legs.** All tibiae with short setae arranged in rows. Mid tibia with four long anterodorsal and ca. 20 short posterodorsal bristles. Hind tibia with eight anterodorsal and five posterodorsal bristles. **Wings.** Sc short, ending in R. Length of rm equal to stem of posterior fork. Base of anterior fork well before base of posterior fork. R1 with setae, R5 without setae. **Male terminalia.** Tergite IX medially divided, each part rounded covered with minute trichia, with one strong apical bristle. Hypandrial lobe heavily sclerotised, elongated and pointed inwards. Gonostylus with dorsal lobe apically tapering; outer surface with several strong setae, in particular near basis. Median lobe flat and broad, larger than dorsal lobe, but more weakly sclerotised; with mixture of short and long setae along posterior edge, some setae on inner surface of lobe apically. Ventral lobe short, club-shaped; with three strong setae apically. Basal part large, with straight edge, lined with strong setae. Internal part with long, caudally projecting process.

#### Etymology.

From Greek, *dibolia*, a two-edged lance, referring to the shape of the dorsal lobe of the gonostylus, which is spear-like.

#### Remarks.

*Allodiadibolia* is very distinct, and the gonostylus clearly separates it from other species of the genus. Superficially the terminalia can resemble those of *A.lugens* (Wiedemann, 1817), with a distinctly pointed dorsal lobe of the gonostylus. *Allodiadibolia*, however, the inner lobe of the gonostylus possess a projecting process, which is lacking in *A.lugens*. The medial lobe is large and broad, not pointed, with many setae, not only along the edge.

### Allodia shimai

Taxon classificationAnimaliaDipteraMycetophilidae

Magnussen sp. n.

http://zoobank.org/64F2EC2B-EA9F-46AD-989B-187C7A964D98

Fig. 2C

#### Diagnosis.

The dorsal lobe of the gonostylus is heavily sclerotised, dark and with numerous strong setae. The median lobe is distinctly square-shaped with four strong bristles at the edge. *Allodiashimai* is a dark species.

#### Type locality.

**NEPAL**: Province no. 1 (Kosi Zone), Sankhuwasabha District, Thudam, 3500–3800 m a.s.l.

#### Type specimens.

***Holotype*** : male. 3 printed labels: (**E. NEPAL**) Thudam (3500-3800m) 27°45'N, 87°31'E---27°46'N, 87°33'E / June 27, 1972 H. Shima leg. Kyushu Univ. Col. / TSZD-JKJ-104961. (KUEC, pinned with genitalia in separate microvial). ***Paratypes***: **NEPAL**: Same data as for holotype except 24 Jun 1972, TSZD-JKJ-104963, male (KUEC); Tanga La, 27°40'N, 087°36'E, 4000–4600 m a.s.l, 6 Jul 1972, Leg. H. Shima, TSZD-JKJ-104962, male (TMU).

#### Description.

Body length 2.5–4.4 mm; wing length 2.7–3.9 mm. **Colouration.** Head and clypeus dark brown. Mouthparts and palpomeres dark yellow. Antennae brown, with scape, pedicel and basis of first flagellomere yellow. Scutum dark brown. Lateral sclerites dark brown, except for yellow antepronotum. Wings clear without markings. Halteres whitish yellow. Legs yellow. Abdomen dark brown. Terminalia yellow. **Head.** Three ocelli present, median ocellus smaller than laterals, lateral ocelli touching eye margin. Head covered with fine trichia, making a silvery appearance, several larger setae in row above eye margin. Antennae approx. twice as long as thorax. Scape with several setae dorsally, pedicel with several setae on distal third. Flagellomeres cylindrical, densely clothed with fine trichia. First flagellomere almost twice as long as scape. **Thorax.** Antepronotum with two long and two shorter setae. Scutum covered with uniform small, pale setae; strong prealar and postalar setae. Few medioposterior discal bristles present. Scutellum with two strong bristles. Laterotergite with four strong setae. Other lateral sclerites bare. **Legs.** All tibiae with short setae arranged in rows. Mid tibia with five long anterodorsal and numerous short posterodorsal bristles. Hind tibia with seven anterodorsal and four posterodorsal bristles. **Wings.** Sc short, ending in R. Length of rm more than double the length of stem of posterior fork. Base of anterior fork opposite base of posterior fork. R1 with setae, R5 without. **Male terminalia.** Tergite IX medially divided, each part rounded, covered with minute trichia, with one strong apical bristle several small setae. Hypandrial lobe heavily sclerotised and elongated. Gonostylus with dorsal lobe prominent, broad, evenly sloping towards slightly prolonged posteroventral corner; outer surface with numerous setae, denser towards basis. Median lobe of gonostylus short, almost squared, with four strong setae along posterior margin and one smaller seta on ventral edge. Ventral lobe of gonostylus club-shaped, pointing ventrally, with some setae apically. Basal part of gonostylus rounded, with many pronounced, thick, short setae. Internal part of gonostylus with long, caudally projecting process.

#### Etymology.

Named after Hiroshi Shima, the collector of the type specimens.

#### Remarks.

The dorsal lobe of the gonostylus is somewhat similar to *A.scalprata*, but much more prominent and larger. Additionally, in *A.shimai* the internal part of the gonostylus possess a caudally projecting process, which is lacking in *A.scalprata*, and the median lobe is prominently sclerotised and unlike that in other *Allodia* species.

### Allodia spathulata

Taxon classificationAnimaliaDipteraMycetophilidae

Magnussen sp. n.

http://zoobank.org/CA4DC3CE-03D0-4C7E-8D8B-6A31CED055FA

Fig. 2D

#### Diagnosis.

The dorsal lobe of the gonostylus is rather narrow and spatula-shaped, and the basal part of the gonostylus has a very distinct, elongate protuberance. This outline of the gonostylus makes this species distinct. *Allodiaspathulata* is a small species (2.2 mm).

#### Type locality.

**NEPAL**: Gandaki Pradesh, Myagdi District, Dobang Kharka, 2400 m a.s.l.

#### Type specimens.

***Holotype*** : male. 2 printed labels: (**NEPAL**) Dobang Kharka, 2400m 083°24'E. 28°36'N. Malaise trap. Oct. 26–27. 1971. A. Nakanishi / TSZD-JKJ-104964 (KUEC, pinned with genitalia in separate microvial). ***Paratype***: **NEPAL**: Province no. 1 (Kosi Zone), Sankhuwasabha District, NE of Thudam, 27°47'N, 087°36'E, 4000 m a.s.l, 30 Jun 1972, Leg. H. Shima, TSZD-JKJ-104965, male (TMU).

#### Description.

Body length 2.2 mm; wing length 2.3 mm. **Colouration.** Head and clypeus brown. Mouthparts and palpomeres yellow. Antennae brown, with scape, pedicel, and basal half of first flagellomere yellow. Scutum light brown, with broad yellow lateral area, from humerus towards wing base. Lateral sclerites light brown, except for yellow antepronotum. Wings clear without markings. Halteres whitish yellow. Legs light yellow. Abdomen brown, with tergites II-IV with lateral yellow area, broadening towards posterior margin. Terminalia yellow. **Head.** Three ocelli present, median ocellus smaller than laterals, lateral ocelli touching eye margin. Head covered with fine trichia, except for four thin setae near eye margin. Antennae approx. twice as long as thorax. Scape and pedicel with several setae on distal third. Flagellomeres cylindrical, densely clothed with fine trichia. First flagellomere almost twice as long as pedicel. **Thorax.** Antepronotum with four strong setae. Scutum covered with uniform small, pale setae; strong prealar and postalar setae. Discal bristles absent. Scutellum with two strong bristles. Laterotergite with five minute setae. Other lateral sclerites bare. **Legs.** All tibiae with short setae arranged in rows. Mid tibia with six long anterodorsal and numerous short posterodorsal bristles. Hind tibia with five anterodorsal and four posterodorsal bristles. **Wings.** Sc short, ending in R. Length of rm slightly shorter than stem of posterior fork. Base of anterior fork opposite base of posterior fork. R1 with setae, R5 without. **Male terminalia.** Tergite IX medially divided, each part rounded, covered with minute tricha, with one strong apical bristle and several small setae. Hypandrial lobe elongated. Gonostylus with dorsal lobe spatula-shaped, with several setae on outer surface; posterior border slightly concave. Median lobe broad, sub-quadrate with several long setae along posterior border, distinctly pointed posterodorsally. Ventral lobe of gonostylus elongated, club-shaped, with four setae apically. Basal part of gonostylus with distinct elongated protuberance, about half the length of median lobe; with four setae apically. Internal part of gonostylus with long, caudally projecting spike.

#### Etymology.

From Latin *spathulatus*, spatula-like, referring to outline of the dorsal lobe of the gonostylus.

#### Remarks.

Only two specimens of this species are present in the material and the terminalia were fragile and difficult to dissect. Several setae of the gonostylus were therefore lost in the dissection, but their bases are depicted (Fig. 2D). The species is superficially similar to *A.zaitzevi* Kurina, 1997 and *A.pyxidiiformis* Zaitzev, 1983, but the dorsal lobe of the gonostylus is much more flattened, forming a distinct spatula-shape with pronounced edges. Compared to other Himalayan species, it most closely resembles *A.horologia*, but this species has a less pronounced protuberance in the basal part of the gonostylus, and the dorsal lobe is broader and more heavily sclerotised.

### Allodia horologia

Taxon classificationAnimaliaDipteraMycetophilidae

Magnussen sp. n.

http://zoobank.org/F249E52B-FB6C-4A47-A48C-2D52E05CD08B

Fig. 2E

#### Diagnosis.

The dorsal lobe of the gonostylus has a distinct hourglass-shape. The outline of the median lobe, with a pointed posterodorsal tip and a rounded posteroventral corner, makes it different from that in other species described here.

#### Type locality.

**NEPAL**: Province no. 1 (Kosi Zone), Sankhuwasabha District, Topke Gola to Thurukba, 2600–3700 m a.s.l.

#### Type specimen.

Holotype: male. 3 printed labels: (E. NEPAL) Topke Gola (3700 m) 27°38'N, 087°35'E--- Thurukba (3700 m) 27°38'N, 087°35'E / July 9, 1972 Pemba Norbu leg. Kyushu Univ. Col. / TSZD-JKJ-104943 (KUEC, pinned with genitalia in separate microvial).

#### Description.

Body length ca. 3 mm; wing length 2.7 mm. Colouration. Head and clypeus brown. Mouthparts brown, palpomeres yellow. Antennae brown, with scape and pedicel, and basal half of first flagellomere yellow. Scutum brown, with yellow lateral area, from humerus towards wing base. Antepronotum yellow, other lateral sclerites brown. Wings clear, without markings. Halteres yellow. Legs yellow. Abdomen dark brown. Terminalia dark yellow. Head. Three ocelli present, median ocellus smaller than laterals, lateral ocelli touching eye margin. Head covered with fine trichia. Antennae approx. twice as long as thorax. Scape and pedicel with multiple dorsal setae. Flagellomeres cylindrical, densely clothed with fine trichia. First flagellomere twice as long as pedicel. Thorax. Antepronotum with four setae. Scutum covered with uniform, small, pale setae; strong prealar and postalar setae. Discal bristles absent. Scutellum with two strong bristles. Laterotergite with few short setae. Other lateral sclerites bare. Legs. All tibiae with short setae arranged in rows. Mid tibia with six long anterodorsal and 15 short posterodorsal bristles. Hind tibia with six long anterodorsal and six long posterodorsal bristles. Wings. Sc short, ending in R. Length of rm equal to stem of posterior fork. R1 with setae, R5 without setae. Male terminalia. Tergite IX medially divided, each part rounded, covered with minute trichia, two strong apical bristles. Hypandrial lobe heavily sclerotised and elongated. Gonostylus with dorsal lobe heavily sclerotised, flattened and hourglass-shaped with multiple outer setae near basis of lobe. Median lobe of gonostylus distinctly pointed posterodorsally, and with rounded posteroventral part; dorsal edge slightly sigmoid; several long setae on posterior margin, two short setae on internal surface of lobe. Ventral lobe club-shaped, with four long setae sub-apically. Basal part of gonostylus with projecting protuberance with several long setae apically. Internal part of gonostylus with long, caudally projecting process.

#### Etymology.

From Latin *horologium*, timepiece, i.e. a device to show progress of time; here referring to the outline of the dorsal lobe of the gonostylus having the shape of a hourglass.

#### Remarks.

*Allodiahorologia* is morphologically similar to *A.spathulata*, but can be separated based on the shape of the median lobe, see further remarks under *A.spathulata*.

### Allodia himalayensis

Taxon classificationAnimaliaDipteraMycetophilidae

Magnussen sp. n.

http://zoobank.org/2A765559-DB82-45AC-97B9-3CAFFFDE94E4

Fig. 3A

#### Diagnosis.

The narrow, elongated dorsal lobe of the gonostylus, together with the triangular-shaped median lobe makes this species separate from other described *Allodia* species. In addition, the basal part of the gonostylus is thick and slightly elongated.

**Type locality**. **NEPAL**: Province no. 1 (Sagarmatha Zone), Solukhumbu District, Tragdobuk, 2950–3180 m a.s.l.

**Type specimens. *Holotype***: male. 2 printed labels: (**E. NEPAL**) Tragdobuk (2950–3180m) 15. viii. 1981 J. Emoto leg. / TSZD-JKJ-104953 (KUEC, pinned with genitalia in separate microvial). ***Paratypes***: **NEPAL**: Same data as for holotype, TSZD-JKJ-104956, male (TMU); **NEPAL**: Province no. 1 (Kosi Zone), Sankhuwasabha District, Salpa La, 27°27'N, 086°55'E, 3000–3200 m a.s.l, 23 Jul 1981, Leg. J. Emoto, TSZD-JKJ-104954, male (KUEC), TSZD-JKJ-104955, male (KUEC); **NEPAL**: Province no. 3 (Sagarmatha Zone), Junbesi Khola, 27°36'N, 086°33'E, 3400–3500 m a.s.l, 12 Aug 1981, Leg. J. Emoto, TSZD-JKJ-104957, male (KUEC).

#### Description.

Body length 3–3.3 mm; wing length 2.6–3.0 mm. **Colouration.** Head and clypeus brown. Mouthparts and palpomeres yellow. Antennae brown, with scape, pedicel and basal half of first flagellomere yellow. Scutum brown, with broad yellow lateral area, from humerus towards wing base. Lateral sclerites brown. Wings clear without markings. Halteres yellow. Legs yellow. Abdomen brown, tergites II-IV with yellow lateral area, larger towards posterior margin. Terminalia yellow. **Head**. Three ocelli present, median ocellus smaller than laterals, lateral ocelli touching eye margin. Head covered with fine trichia, except for longer setae near eye margin and between eyes. Antennae almost twice as long as thorax. Scape and pedicel with several setae on distal third. Flagellomeres cylindrical, densely clothed with fine trichia. First flagellomere approx. twice as long as pedicel. **Thorax**. Antepronotum with five long setae. Scutum covered with uniform small, pale setae; strong prealar and postalar setae. Discal bristles absent. Scutellum with two strong bristles. Lateral sclerites bare. **Legs**. All tibiae with short setae arranged in rows. Mid tibia with four long anterodorsal and 23 short posterodorsal bristles. Hind tibia with five strong anterodorsal and six strong posterodorsal bristles. **Wings**. Sc short, ending in R. Length of rm almost twice as long as length of posterior fork. Base of anterior fork before base of posterior fork. R1 and R5 with setae. **Male terminalia**. Tergite IX medially divided, each part rounded, covered with minute trichia, one short and one long apical bristle. Hypandrial lobe heavily sclerotised and elongated. Gonostylus with dorsal lobe straight, long and slender, posteriorly blunt, pointed posterodorsally; outer surface with multiple long setae. Median lobe sub-triangular, posterior margin slightly serrated with several long and few shorter setae, distinctly prolonged porsterodorsally; ventral margin with one long seta. Ventral lobe of gonostylus nearly club-shaped, with several long setae apically. Basal part of gonostylus large, rounded, with broad protuberance bearing numerous short setae distally. Internal part of gonostylus without projecting process.

**Figure 3.**
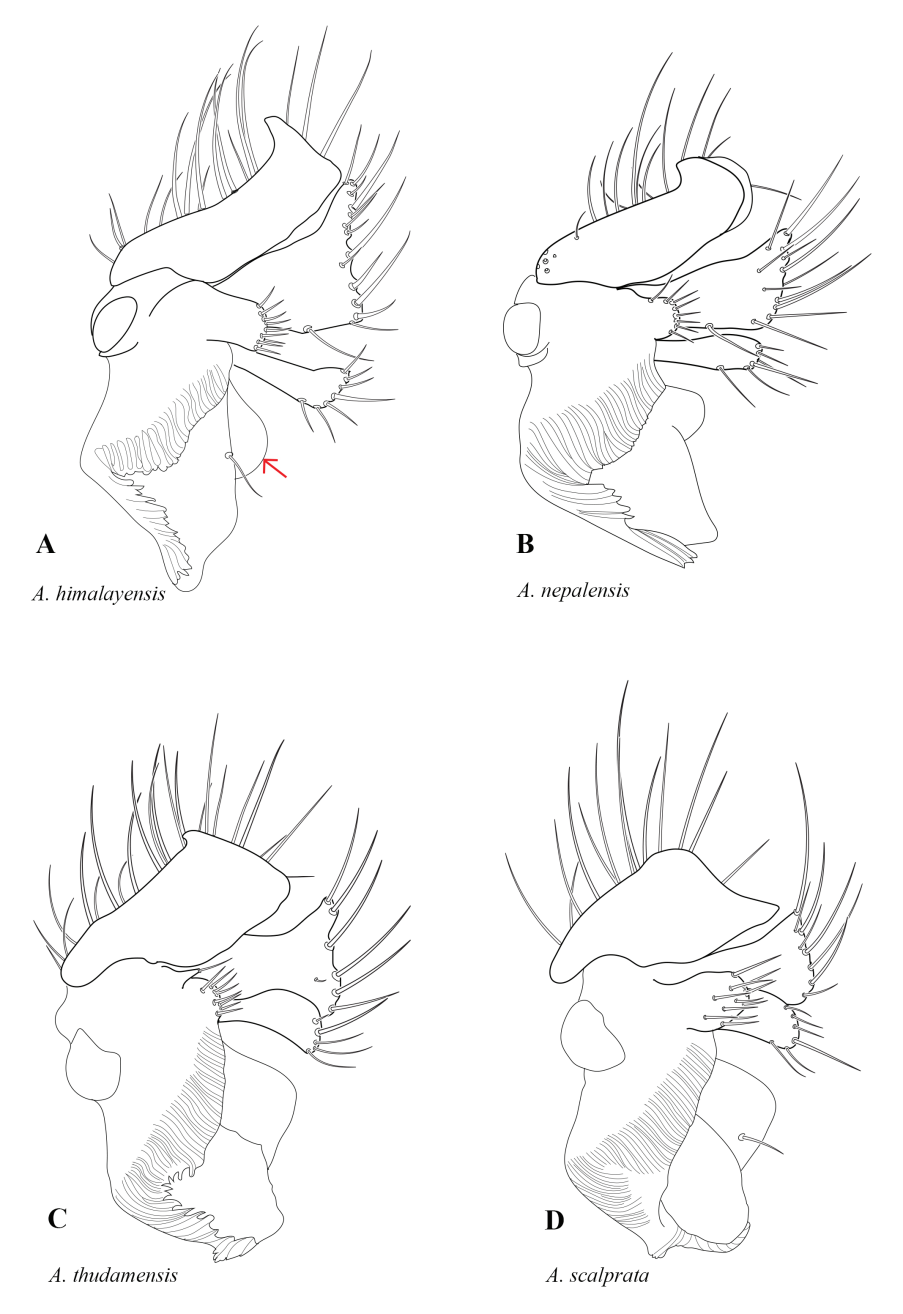
Gonostyli from inner side. **A***Allodiahimalayensis***B***Allodianepalensis***C***Allodiathudamensis***D***Allodiascalprata*. Red arrow indicates the absence of a caudally projecting process of the internal lobe.

#### Etymology.

Named after the Himalayan mountain region of the type locality.

**Remarks.***Allodiahimalayensis* is superficially similar to *A.thudamensis*, but the dorsal lobe of the gonostylus is much more elongated, and narrower, than in *A.himalayensis*. There is also a certain similarity to *A.ornaticollis* (Meigen, 1818), but *A.ornaticollis* has a projecting spike from the internal part of the gonostylus, which is absent in *A.himalayensis*.

### Allodia nepalensis

Taxon classificationAnimaliaDipteraMycetophilidae

Magnussen sp. n.

http://zoobank.org/61117AC7-B15B-4B23-BD73-8937D5957EBC

Fig. 3B

#### Diagnosis.

The dorsal lobe of the gonostylus is posteroventrally rounded, with an apical, membranous pouch-like structure. The median lobe is narrow, with several long setae, also on the surface of the lobe. In combination these characters make this species distinct, compared to other *Allodia* species.

#### Type locality.

**NEPAL**: Province no. 1 (Kosi Zone), Sankhuwasabha District, Thudam, 3500–3800 m a.s.l.

#### Type specimen.

***Holotype:*** male. 3 printed labels: (**E. NEPAL**) Thudam (3500–3800m) 27°45'N, 87°31'E---27°46'N, 87°33'E / June 24, 1972 H. Shima leg. Kyushu Univ. Col. / TSZD-JKJ-104958 (KUEC, pinned, with genitalia in separate microvial).

#### Description.

Body length ca. 3 mm; wing length 3.24 mm. **Colouration.** Head and clypeus dark brown. Mouthparts and palpomeres brown. Antennae brown, with pedicel yellow. Scutum brown. Lateral sclerites brown. Wings clear without markings. Halteres whitish yellow. Legs yellow. Abdomen brown. Terminalia yellow. **Head.** Three ocelli present, median ocellus smaller than laterals, lateral ocelli touching eye margin. Head covered with fine trichia, several longer setae above eye margin. Antennae ca. 1.5 times longer than thorax. Scape and pedicel with several small setae dorsally. Flagellomeres cylindrical, densely clothed with fine trichia. First flagellomere almost twice as long as pedicel. **Thorax.** Antepronotum with four long setae. Scutum covered with small, pale setae; few, strong prealar and postalar setae. Discal bristles absent. Scutellum with two strong bristles. Laterotergite with two strong and four small setae. Other sclerites bare. **Legs.** All tibiae with short setae arranged in rows. Mid tibia with five and approximately 15 short posterodorsal bristles. Hind tibia with seven anterodorsal and five posterodorsal bristles. **Wings.** Sc short, ending in R. Length of rm twice as long as length of stem of posterior fork. Base of anterior fork opposite base of posterior fork. R1 and R5 with setae. **Male terminalia.** Tergite IX medially divided, each part rounded, covered with minute trichia, with one long and one short apical bristle. Hypandrial lobe sclerotzed and elongated. Gonostylus with dorsal lobe elongated, posteriorly rounded with small upturned hook; outer surface of lobe with numerous long setae, membranous pouch-like structure present apically. Median lobe rather narrow, broadening towards apex; several long setae on posterior edge, short setae also present on internal surface of lobe apically; ventral lobe of gonostylus nearly club-shaped, with one ventral seta, and several posterior setae. Basal part of gonostylus rounded, with numerous short setae. Internal part of gonostylus without projecting process.

#### Etymology.

From Nepal, and Latin ending –*ensis*, belonging to Nepal.

#### Remarks.

Superficially the male terminalia resemble those of *A.himalayensis*, but the dorsal lobe of the gonostylus possesses a membranous, pouch-like structure apically, which is lacking in *A.himalayensis*.

### Allodia thudamensis

Taxon classificationAnimaliaDipteraMycetophilidae

Magnussen sp. n.

http://zoobank.org/BC9A90B5-97F1-420F-9468-9E63CCFBA772

Fig. 3C

#### Diagnosis.

The broad and short dorsal lobe of the gonostylus, possessing a slight hook posterodorsally makes this species distinct. In addition, the median lobe of the gonostylus has six long setae along the posterior edge.

#### Type locality.

**NEPAL**: Province no. 1 (Kosi Zone), Sankhuwasabha District, Thudam, 3500–3800 m a.s.l.

#### Type specimens.

***Holotype*** : male. 3 printed labels: (**E. NEPAL**) Thudam (3500–3800 m) 27°45'N, 87°31'E---27°46'N, 87°33'E / June 24, 1972 H. Shima leg. Kyushu Univ. Col. / TSZD-JKJ-104966, male (KUEC, pinned with genitalia in separate microvial).

#### Description.

Body length ca. 3.4 mm; wing length 3.3 mm. **Colouration.** Head and clypeus dark brown. Mouthparts and palpomeres yellow. Antennae brown, with scape, pedicel, and basis of first flagellomere yellow. Scutum brown. Antepronotum yellow. Other lateral sclerites brown. Wings clear without markings. Halteres whitish. Legs yellow. Abdomen brown. Terminalia yellow. **Head.** Three ocelli present, median ocellus smaller than laterals; lateral ocelli touching eye margin. Head covered with fine trichia. Antennae over twice as long as thorax. Scape and pedicel with small setae on distal third. Flagellomeres cylindrical, densely clothed with fine trichia. First flagellomere twice as long as pedicel. **Thorax.** Antepronotum with five strong setae. Scutum covered with uniform small, pale setae. A few posterior discal bristles present. Scutellum with two strong bristles and few smaller setae. Laterotergite with five setae. Other lateral sclerites brown. **Legs.** All tibiae with short setae arranged in rows. Mid tibia with four anterodorsal and 13 posterodorsal setae. Hind tibia with four anterodorsal and seven posterodorsal setae. **Wings.** Sc short, ending in R. Length of rm slightly longer than stem of posterior fork. Base of anterior fork opposite base of posterior fork. R1 with and R5 without setae. **Male terminalia.** Tergite IX medially divided, each part rounded, covered with minute trichia, with one long and one short apical bristle. Hypandrial lobe heavily sclerotzed and elongated. Gonostylus with dorsal lobe broad, short with flattened apex, with posterodorsal hook; outer surface with numerous long setae. Median lobe of gonostylus sub-triangular with broad, pointed posterodorsal corner; posterior margin with six strong setae, one small seta on surface of lobe. Ventral lobe of gonostylus club-shaped; with five apical setae. Basal part of gonostylus small, rounded; with multiple short setae. Internal part of gonostylus without projecting process.

**Etymology.** From Thudam, the type locality, and Latin ending -*ensis*, belonging to Thudam.

**Remarks.***Allodiathudamensis* resembles two Holarctic species, viz. *A.embla* Hackman, 1971 and *A.anglofennica* Edwards, 1921. It can be separated from both these species by the combination of having a subrectangular outline of the dorsal lobe, as opposed to a more sinusoid, skewed shape found in *A.embla*. A pointed posterodorsal corner of the medial lobe, as opposed to angular cut corner in *A.anglofennica*. Additionally, *A.embla* has numerous setae along the posterior edge of the median lobe of gonostylus, while only six strong setae are present in *A.thudamensis*. The median lobe of the gonostylus is differently shaped in *A.thudamensis* and *A.anglofennica*.

### Allodia scalprata

Taxon classificationAnimaliaDipteraMycetophilidae

Magnussen sp. n.

http://zoobank.org/CBA17A0A-9B0F-449F-B80D-F668737AECDB

Fig. 3D

#### Diagnosis.

The dorsal lobe of the gonostylus is short, but broad, with clear-cut corners. The median lobe is slender, without a prominent posterodorsal tip. The basal part of the gonostylus is distinct and prominently bud-shape, with several strong setae apically.

#### Type locality.

**NEPAL**: Province no. 1 (Kosi Zone), Sankhuwasabha District, Salpa La, 2900–3000 m a.s.l.

**Type specimens. *Holotype***: male. 2 printed labels: (**E. NEPAL**) Salpa La (2900~3000m) 29.vii.1981 J. Emoto / TSZD-JKJ-104959 (KUEC, pinned, with genitalia in separate microvial). ***Paratype***: **NEPAL**: Same data as for holotype, TSZD-JKJ-104960, male (TMU).

#### Description.

Body length 3.0–3.4 mm; wing length 3.0–3.3 mm. **Colouration.** Head and clypeus brown. Mouthparts and palpomeres yellow. Antennae brown, with scape, pedicel and basal half of first flagellomere yellow. Scutum brown, with yellow lateral area, from humerus towards wing base. Antepronotum yellow. Other lateral sclerites brown. Wings clear without markings. Halteres whitish. Legs yellow. Abdomen brown. Terminalia yellow. **Head.** Three ocelli present, median ocellus smaller than laterals; lateral ocelli touching eye margin. Head covered with fine trichia. Antennae approx twice as long as thorax. Scape and pedicel with several setae at apex. Flagellomeres cylindrical, densely clothed with fine trichia. First flagellomere twice as long as pedicel. **Thorax.** Antepronotum with six strong setae. Scutum covered with uniform small, pale setae; strong prealar and postalar setae. Few posterior discal bristles present. Scutellum with two strong bristles, and several short setae. Laterotergite with five setae. Other lateral sclerites bare. **Legs.** All tibiae with short setae arranged in rows. Mid tibia with five long anterodorsal and numerous short posterodorsal setae. Hind tibia with seven long anterodorsal and seven long posterodorsal setae. **Wings.** Sc short, ending in R. Length of rm approx. twice as long as stem of posterior fork. Base of anterior fork just before base of posterior fork. **Male terminalia.** Tergite IX medially divided, each part rounded, covered with minute trichia, one short and one long apical bristle. Hypandrial lobe heavily sclerotised and elongated. Gonostylus with short dorsal lobe, with blunt posterodorsal corner and sharp, edgy posteroventral corner; multiple long setae on outer surface of lobe. Median lobe oblong and narrow, posterodorsal tip not prominent; with five long and three shorter setae on posterior edge. Ventral lobe nearly club-shaped, broad, apically with five small and one long setae. Basal part of gonostylus prominent, rounded, with slightly constricted basis, with several strong, but short setae apically. Internal branch of gonostylus without projecting process.

#### Etymology.

From Latin *scalprum*, a knife or chisel, referring to the clean edge of the dorsal lobe of the gonostylus.

#### Remarks.

*Allodiascalprata* is morphologically similar to *A.shimai*, and also to *A.nepalensis*. The dorsal lobe of the gonostylus is similar to *A.shimai*, but shorter and not so prominent. Additionally, *A.shimai* possesses a projecting process of the internal part of the gonostylus, which is lacking in *A.scalprata*. *Allodiascalprata* can also superficially resemble *A.nepalensis*, due to the short and broad dorsal lobe of the gonostylus, but the dorsal corner is differently shaped in the two species.

## Discussion

The descriptions of the new species here presented are all founded on male holotypes, and considered as distinct species with good diagnostic characters in the outline of the male genitalia. They have been thoroughly compared to other known species of *Allodia* in the Holarctic and Oriental region, and found not to be conspecific to any of these. For the majority of species, including all species with a Holarctic distribution, this assumption is, above all, based on the distinct differences in the outline of the male genitalia.

Three species of *Allodia* are listed in “Catalog of the Diptera in the Oriental region” (Colless & Liepa 1973) *A.micans* Edwards, 1928 from Malaysia, *A.nigrofasciata* Brunetti, 1912 from northern India, and *A.varicornis* (Senior-White, 1922) from Sri Lanka. The description of *A.micans* and *A.varicornis* were both based on females, but we exclude the possibility that they could be conspecific with any of the new species as we find it improbable that their distribution will include the Himalayas. Moreover, details in the original descriptions of the two species, like the colouration of the abdomen, make it likely that the two belong to the subgenusBrachycampta.

According to the known distribution, *Allodianigrofasciata* is a more likely candidate to be found in the Himalayas. Brunetti (1912) based his description on type specimens from Shimla in the Himachal Pradesh district, but a female was also recorded from Mundali, Dehradun, in the Uttarakand district. We have not studied type material of this species as Brunetti's type material is today at best considered inaccessible (see e.g., Väisänen 1996). Edwards (1924) was given the opportunity to re-examine material of ca. 50 species described by Brunetti from India, but regrettably, *A.nigrofasciata* was not among those. After thoroughly reviewing the description of this species, we find it justified to transfer this species to the genus *Brevicornu* Marshall, 1896. The new combination, *Brevicornunigrofasciatum* (Brunetti, 1912) comb. n., is based on the original description, including a line drawing of the wing. The illustrated wing venation with a rather sessile posterior fork, the description of small thoracic bristles intermingled among soft pale hairs, indicates *Brevicornu* as a more appropriate genus. Additionally, in the description of the male terminalia Brunetti (1912: 108) states that “the large basal joint of each clasper terminating in two narrow flexible finger-like appendages, and between the claspers a pair of branched black hook-like organs”, which is a very strong indication that the species belongs to the genus *Brevicornu* Marshall, 1896 sensu Tuomikoski (1966). One should note, however, that at the time of Brunetti's work, *Brevicornu* was considered to be congeneric with *Allodia* s. l. (cf. Johannsen 1909).

More recently, Wu et al. (2003) have described seven *Allodia* species from the Oriental region. The material is collected in the provinces Zheijiang, Guangxi, and Guizhou in SE China, which are geographically distant from the Himalayas. The descriptions are accompanied by adequate line drawings, and we have studied the descriptions thoroughly. Despite questioning the affiliation to genera for some of the species, we definitely conclude that none of the seven species described by Wu et al. (2003) are conspecific with the species described here.

This study has demonstrated a high species diversity in material consisting of a relatively small number of specimens. In the studied material no described species were found, which also indicates a high uniqueness of taxa in the area. This clearly emphasises the fact that the fauna of fungus gnats in the Himalayas still undoubtedly covers many undescribed and unrecorded species, and that more studies are needed to reveal the full extent of the diversity in this area.

## Supplementary Material

XML Treatment for Allodia caligata

XML Treatment for Allodia dibolia

XML Treatment for Allodia shimai

XML Treatment for Allodia spathulata

XML Treatment for Allodia horologia

XML Treatment for Allodia himalayensis

XML Treatment for Allodia nepalensis

XML Treatment for Allodia thudamensis

XML Treatment for Allodia scalprata
